# Reflective functioning in mothers with addictions: Differential relationships involving family history of mental illness and substance use

**DOI:** 10.3389/fpsyg.2022.911069

**Published:** 2022-10-13

**Authors:** Amanda F. Lowell, Tal Yatziv, Elizabeth Peacock-Chambers, Amanda Zayde, Cindy DeCoste, Nancy Suchman, Thomas J. McMahon

**Affiliations:** ^1^Child Study Center, School of Medicine, Yale University, New Haven, CT, United States; ^2^Department of Pediatrics, University of Massachusetts Chan Medical School-Baystate, Springfield, MA, United States; ^3^Montefiore Medical Center, Bronx, NY, United States; ^4^Albert Einstein College of Medicine, New York, NY, United States; ^5^Department of Psychiatry, School of Medicine, Yale University, New Haven, CT, United States

**Keywords:** maternal addiction, reflective functioning, family history, attachment trauma, early adversity

## Abstract

Parental reflective functioning (RF) is often cited as an important domain in which mothers with addictions struggle in their roles as parents, though the links between addiction and RF remain unclear. Exposure to attachment trauma associated with parental mental illness and substance use is commonly associated with both addiction and lower RF. We thus examined how family history of parental mental illness and substance use may relate to the RF of mothers with addictions. One hundred ninety-four mothers in outpatient substance use treatment completed the Parent Development Interview and provided information about whether their mothers and fathers experienced mental illness or problems with substance use. Univariate ANOVAs revealed an interaction between family history of maternal mental illness and maternal substance use. Among mothers with a history of maternal substance use, those with a history of maternal mental illness had higher RF than those who had no history of maternal mental illness. Among mothers who did not report a family history of maternal mental illness, mothers who had a family history of maternal substance use exhibited significantly lower RF than mothers with no family history of maternal substance use. Exposure to paternal mental illness or substance use was not associated with mothers’ RF. These findings highlight the importance of disentangling the contributions of attachment trauma to mothers’ RF and utilizing interventions that support mothers’ capacity to reflect about how their early experiences of being cared for by a mother with a mental illness or addiction may impact their current caregiving behaviors.

## Introduction

Mothers with addictions are often described in the literature as experiencing difficulties in their roles as parents. Firsthand accounts from mothers experiencing addiction describe challenges balancing recovery with caring for young children (Gazso, 2021), as well as feelings of ambivalence and guilt around the intersection of parenthood and substance use (Silva et al., 2013). Numerous empirical studies document that during interactions with their children, mothers with substance use disorders demonstrate poorer sensitivity to their children’s signals (Hatzis et al., 2017; Porreca et al., 2018; Romanowicz et al., 2019) as well as more harsh parenting practices (Kelley et al., 2015) and high rates of involvement with child welfare and legal systems (Paltrow and Flavin, 2013; Taplin and Mattick, 2015; Moreland et al., 2021). Poor parental *reflective functioning* (RF), or the capacity to make sense of one’s own and one’s child’s behaviors in terms of thoughts, emotions, wishes, and intentions (Slade, 2005), underlies many of these caregiving challenges (Suchman et al., 2004; Pajulo et al., 2006; Suchman et al., 2006; Macfie et al., 2020).

Notably, one’s level of RF originates within early attachment relationships. Ideally, infants are thought to learn about the minds of others by being held in mind by their caregivers (Fonagy and Target, 1997; Slade, 2005). However, caregiving contexts in which this process is disrupted may impede the development of RF capacities. Examining the caregiving contexts experienced by mothers with addictions may therefore be important in order to better understand what may be at play in the manifestation of their RF as they care for the next generation.

Parental mental illness and substance use in one’s family growing up are two such caregiving contexts in which mothers may not have consistently experienced the opportunity to develop optimal RF. Specifically, parental mental illness and substance use may result in *attachment trauma* (Allen, 2018), the child’s experience of the caregiver as physically or emotionally unavailable to support and regulate them when experiencing distress, further contributing to the child’s interpersonal distress and distrust. In the case of parental mental illness, a caregiver’s emotional lability, irritability, under-regulation, affective distress, or inconsistency can be frightening to the infant who is in need of comfort. In the case of parental substance use, a caregiver’s detachment, intoxication, withdrawal symptoms, and preoccupation with obtaining substances can be similarly distressing to the infant who depends on activating and engaging the caregiver for security and survival. Given that “understanding of minds is hard without the experience of having been understood as a person with a mind” (Fonagy and Target, 2005, p. 334), we might anticipate that individuals whose caregivers were misattuned to their minds and emotions because of mental illness or substance use may grow into adults, have children, and struggle in their parental RF, with the potential for repeating maladaptive intergenerational patterns. This is evident in prior work demonstrating that, for mothers with addictions in particular, those who demonstrated poor RF had experienced significantly more adversity in childhood compared to mothers with moderate to high RF (Håkansson et al., 2018).

In this study, we thus sought to identify how specific risk factors for attachment trauma, namely family history of maternal and paternal mental illness and substance use, may relate to the parental RF of mothers with addictions. Given evidence that attachment representations of maternal and paternal figures evolve independently with the potential for differential effects on developmental outcomes for the child (Dagan and Sagi-Schwartz, 2018; Dagan et al., 2022), we predicted that a maternal history of mental illness and substance use would both be associated with mothers’ RF. Similarly, we predicted that a paternal history of mental illness and substance use would also both be associated with RF. Consistent with prior research (Macfie et al., 2020) and cumulative risk theory (Evans et al., 2013) wherein multiple exposures to adversity are associated with poorer outcomes, we also predicted that mothers in our sample who experienced both a family history of mental illness and substance use would exhibit lower RF compared to mothers who experienced parental mental illness or parental substance use alone. Specifically, (a) mothers with no exposure to either parental mental illness or parental substance use would have greater RF than mothers exposed to either alone and (b) mothers exposed to either parental mental illness or parental substance use alone would have greater RF than mothers exposed to both. Such findings would have the potential to inform intervention efforts aimed at improving the RF of mothers with addictions.

## Methods

### Participants

In the current study, we used baseline data from 194 mothers recruited across three randomized controlled trials (RCT) testing the efficacy of a parenting intervention designed specifically for mothers in treatment for substance use disorders: *Mothering from the Inside Out* (Suchman et al., 2008, 2010a,^2011^, ^2017^; Lowell et al., 2022). Mothers were recruited from a community-based outpatient substance use treatment agency comprised of several clinics in the greater New Haven, CT area. Mothers were referred to participate in this research by substance use counselors, research staff, or word of mouth.

Mothers were eligible to participate in the study if they were enrolled in outpatient substance use treatment, English-speaking, and caring for at least one child between birth and 60 months of age at time of enrollment. If mothers had more than one child in their care within this age range, mothers chose which child they wanted to focus on when completing the parenting-focused research measures. Exclusion criteria included mothers requiring inpatient hospitalization or detoxification, and mothers experiencing psychosis, suicidality, or significant cognitive impairment. Mothers who met eligibility requirements met individually with a research assistant to complete informed consent procedures.

Baseline data for the first (*n* = 43), second (*n* = 68), and third (*n* = 83) RCTs were merged into a single dataset to increase sample size and statistical power. On average, mothers were approximately 30 years old, had a high school education, and had an average of 2 children. Most mothers were white, unemployed, and were on medication for an opioid use disorder (MOUD). Mothers generally began using substances at a young age, and reported low levels of substance use in the past month. Detailed demographic data can be found in Table 1.

**TABLE 1:** Demographic characteristics by trial.

	RCT 1 (*n* = 43)	RCT 2 (*n* = 68)	RCT 3 (*n* = 83)	Total (*N* = 194)	Range
	
	M (SD)	M (SD)	M (SD)	M (SD)	
Age (years)	29.23 (6.50)	29.96 (5.22)	30.89 (4.04)	30.20 (5.10)	19–45
Child age (months)*	23.67 (9.08)	29.36 (15.27)	31.42 (14.38)	29.46 (14.24)	9–59
Number of biological children	2.02 (1.14)	2.04 (1.19)	1.96 (1.08)	2.01 (1.13)	1–6
Education (years)	12.44 (1.28)	12.59 (2.16)	12.95 (1.90)	12.71 (1.89)	8–18
Employment					
Employed	18.6%	20.6%	28.9%	23.7%	
Unemployed	81.4%	79.4%	71.1%	76.3%	
Ethnicity					
White	67.4%	76.5%	77.1%	74.7%	
Black	25.6%	14.7%	8.4%	14.4%	
Hispanic/Latina	7.0%	2.9%	8.4%	6.2%	
Other/Multiple	0.0%	5.9%	6.0%	4.6%	
Marital status					
Never married	55.8%	38.2%	36.1%	41.2%	
Married/Cohabitating	27.9%	52.9%	56.6%	49.0%	
Separated/Divorced	16.3%	8.8%	7.2%	9.8%	
Child welfare involvement**					
Yes	58.1%	30.9%	31.1%	37.1%	
No	41.9%	69.1%	68.7%	62.9%	
MOUD					
Yes	65.1%	69.1%	75.9%	71.1%	
No	34.9%	30.9%	24.1%	28.9%	
Substance use in past month (days)					
Alcohol	0.48 (1.38)	1.02 (2.43)	1.06 (3.08)	0.93 (2.47)	0–18
Cannabis**	0.13 (0.40)	1.38 (4.66)	2.74 (6.63)	2.04 (6.38)	0–30
Cocaine	0.18 (0.81)	0.44 (2.03)	0.20 (0.81)	0.27 (1.34)	0–15
Heroin	0.25 (1.58)	0.34 (1.21)	0.16 (1.13)	0.23 (1.21)	0–10
Other opioids	0.90 (4.81)	0.00 (0.00)	0.02 (0.14)	0.22 (2.33)	0–30
Age of substance use onset (years)					
Alcohol	13.33 (3.11)	13.97 (2.21)	13.84 (3.06)	14.36 (2.90)	4–21
Cannabis	14.22 (1.44)	13.64 (1.61)	13.88 (2.05)	14.04 (2.03)	8–21
Cocaine	19.56 (4.89)	18.05 (3.28)	18.24 (3.76)	19.04 (4.17)	12–36
Heroin	22.11 (4.54)	20.33 (4.02)	20.98 (4.86)	20.99 (4.75)	12–37
Other opioids**	22.17 (4.97)	18.28 (3.53)	18.66 (4.20)	19.53 (4.80)	13–35

RCT, randomized controlled trial; MOUD, medication for opioid use disorder.

*p < 0.05, **p < 0.01.

### Measures

#### Family history

A 90-min structured intake interview was used to identify baseline demographic and psychosocial characteristics of the sample. Mothers provided information about their psychosocial history including their own substance use, psychiatric, legal, and medical history. They also provided information regarding their early development and family history including whether their mother and father experienced mental health or substance use problems. Specifically, mothers provided responses to items derived from the *Addiction Severity Index* (ASI; McLellan et al., 1992) asking whether any of their biologically-related relatives experienced significant substance use or psychiatric problems that did (or should have) led to treatment. For the current study, we were interested in mothers’ dichotomous yes/no responses to whether their (1) mother experienced mental illness, (2) mother experienced substance use problems, (3) father experienced mental illness, and (4) father experienced substance use problems. The ASI has good reliability and validity in terms of assessing history of first-degree relatives’ substance use and psychiatric problems (Cacciola et al., 1999; Coviello et al., 2004).

#### Reflective functioning

The *Parent Development Interview* (PDI; Slade et al., 2003), a widely used instrument for assessing parental reflective functioning, is a semi-structured interview with 19 questions designed to elicit a parent’s verbal narrative about common emotionally-challenging experiences of caring for young children. With permission from the measure’s originator, a shortened 14-item version was used to minimize assessment burden and include positive emotion items (e.g., “Have you ever felt deeply touched or moved as a parent?”). On each item, additional queries were included to encourage the mother to consider her own and her child’s intentions and emotions during these instances and how these mental states might have affected her and her child’s behavior. Interviews were recorded and transcribed verbatim, and mothers’ responses were coded for parental reflective functioning (Slade et al., 2007) by coders who were trained by the measure’s originator, and who were blind with respect to the timepoint at which the interview was conducted and the clinical characteristics and random assignment of the participants. Each response was rated on a 10-point scale ranging from −1 to 9, where −1 indicates active resistance to reflecting about mental states; 1 indicates the absence of recognition of mental states (i.e., events are described solely in terms of behavior); 3 indicates a limited capacity to acknowledge mental states and a lack of understanding how mental states function; 5 indicates ordinary reflective functioning, with mothers demonstrating a rudimentary capacity to understand how mental states work together and influence behavior; scores above 5 indicate an increasingly rich and complex understanding of how mental states arise, interact within and between individuals, and influence relational behavior. In each of the three trials, the mean of all responses was calculated to generate a mean RF score. Inter-rater reliability was established for each trial, with interclass correlations established as ≥0.50 in the first trial (Suchman et al., 2010a,^2011^), ≥0.77 in the second trial (Suchman et al., 2017), and ≥0.57 in the third trial (Lowell et al., 2022).

### Procedure

The current study employed a cross-sectional approach where all baseline data were obtained during the pre-treatment phase of three RCT cohorts testing the efficacy of *Mothering from the Inside Out*. Baseline assessments consisted of a comprehensive biopsychosocial intake interview, the PDI, and several additional measures that were not used in this study. All procedures were approved by the Yale University Human Investigation Committee.

### Data analysis

Two univariate ANOVAs were conducted with mean RF as the dependent variable. Mothers’ family history of mental illness (yes/no) and substance use (yes/no) and an interaction term were entered as the independent variables. ANOVAs were conducted separately for maternal and paternal history. Four *post hoc* comparisons were conducted for significant interaction effects. The impact of parental substance use within parental mental illness was examined, and the impact of parental mental illness within parental substance use was examined. Effect sizes are presented as partial eta-squared (η*_*p*_*^2^), where 0.01 represents a small effect size, 0.06 a medium effect size, and 0.14 a large effect size (Cohen, 1988).

## Results

### Preliminary analyses

Mothers in the sample generally demonstrated suboptimal reflective functioning (*M* = 3.15, *SD* = 0.54) in light of scoring guidelines where a score of 3 reflects the use of mental state language (happy, sad, etc.) without elaboration upon the meaning of the child’s mental states or evidence of linking mental states to behavior (Slade et al., 2003). Analysis of between group differences in demographic characteristics indicated that mothers across the three RCTs did not differ in terms of age, education, employment status, ethnicity, marital status, enrollment in MOUD, and number of children (*p*’s > 0.11). However, mothers in the first trial reported more involvement with child welfare [*F*_(2,191)_ = 5.45, *p* < 0.005], and earlier onset of opioid use [*F*_(2,141)_ = 6.11, *p* < 0.003] compared to mothers in the second and third trials, as well as younger target children compared to those in the third trial [*F*_(2,174)_ = 3.10, *p* < 0.05]. Additionally, mothers in the third trial reported more cannabis use in the past month compared to mothers in the first trial [*F*_(2,189)_ = 5.37, *p* < 0.005].

Given previous work demonstrating a relationship between parental RF and sociodemographic factors (Sleed et al., 2018), we explored these relationships prior to our main analyses. We found that mean RF was related to maternal age (*r* = 0.16, *p* < 0.03) and education (*r* = 0.16, *p* < 0.02). However, when we controlled for these variables in the analyses, the results did not differ; we therefore report results without covariates included in the statistical model.

### Maternal history

A univariate ANOVA specifying maternal history (mental illness/substance use) was conducted to examine mothers’ mean RF. This analysis revealed a main effect for maternal mental illness [*F*_(1,190)_ = 1.68, MSE = 0.28, *p* = 0.02, η*_*p*_*^2^ = 0.03], a main effect for maternal substance use [*F*_(1,190)_ = 1.68, MSE = 0.28, *p* = 0.02, η*_*p*_*^2^ = 0.03], and a significant interaction between history of maternal mental illness and maternal substance use [*F*_(1,190)_ = 4.24, MSE = 0.28, *p* = 0.04]. Inclusion of mothers’ age and years of education as covariates did not alter the pattern of results.

*Post hoc* comparisons revealed that among mothers who reported a family history of maternal mental illness, maternal substance use was not associated with differences in mean RF [*F* < 1, *p* > 0.05]. In contrast, among mothers who did not report a history of maternal mental illness, mothers who reported a family history of maternal substance use exhibited significantly lower mean RF than mothers with no family history of maternal substance use [*F*_(1,190)_ = 10.33, MSE = 0.28, *p* = 0.002, η*_*p*_*^2^ = 0.05].

Among mothers with a history of maternal substance use, those with a history of maternal mental illness had higher RF than those who had no history of maternal mental illness [*F*_(1,190)_ = 10.65, MSE = 0.28, *p* = 0.001, η*_*p*_*^2^ = 0.05]. Among mothers with no family history of maternal substance use, there was no difference between mothers who did or did not report a family history of maternal mental illness [*F* < 1, *p* > 0.05]. See Table 2 for descriptive statistics.

**TABLE 2:** Descriptive statistics for mothers’ mean RF by family history of maternal mental illness and maternal substance use.

	Maternal substance use			
	No		Yes	
Maternal mental illness	*n*	M (SD)	*n*	M (SD)
No	67	3.20 (0.51)	27	2.81 (0.44)
Yes	25	3.24 (0.66)	75	3.20 (0.51)

### Paternal history

A univariate ANOVA specifying paternal history (mental illness/substance use) was conducted to examine mothers’ mean RF. This analysis revealed no significant main effect of paternal mental illness, no significant main effect of paternal substance use, and no significant interaction effect on mothers’ RF [*F*’s < 1, *p*’s > 0.05]. Because the interaction effect was not significant, no pairwise comparisons were conducted. Inclusion of mothers’ age and years of education as covariates did not alter the pattern of results. See Table 3 for descriptive statistics.

**TABLE 3:** Descriptive statistics for mothers’ mean RF by family history of paternal mental illness and paternal substance use.

	Paternal substance use			
	No		Yes	
Paternal mental illness	*n*	M (SD)	*n*	M (SD)
No	52	3.11 (0.53)	60	3.23 (0.54)
Yes	11	3.31 (0.57)	57	3.09 (0.51)

## Discussion

This study utilized baseline data merged across three RCTs testing the efficacy of *Mothering from the Inside Out*, a parenting intervention designed specifically for mothers in treatment for substance use disorders. A large sample of mothers in outpatient substance use treatment completed the PDI as a measure of RF and provided the opportunity to examine differential relationships involving family history of mental illness and substance use. Overall, our findings replicate and extend prior literature on mothers with addictions suggesting that perceptions of early attachment relationships influence current caregiving (Suchman et al., 2005). Consistent with our first hypothesis, a family history of maternal mental illness and substance use were specifically associated with mothers’ RF, although paternal mental illness and substance use were not. Our cumulative risk hypothesis was not supported given the significant interaction effect where mothers who reported a family history of maternal substance use alone demonstrated lower RF than those who reported both maternal substance use and maternal mental illness. Further, counter to prior work denoting that greater adversity was related to poorer RF (Håkansson et al., 2018), among mothers in our sample who experienced a family history of maternal mental illness, family history of maternal substance use was surprisingly not associated with lower RF. While caution is warranted to not over-interpret these findings, we suggest some possible explanations and clinical implications that will require further investigation.

In the absence of maternal mental illness, our hypothesis that a family history of maternal substance use would be associated with lower RF stood true. This is consistent with literature suggesting that parents with substance use problems may be withdrawn and neglectful (Kepple, 2018), which does not characterize an environment in which a child’s reflective capacity is fostered (Allen, 2018). Further, maternal substance use might be characterized by *psychological unavailability*, which Allen (2018, p. 204) notes, “had a greater adverse impact on development than physical neglect or other forms of maltreatment. Thus the subtlest form of maltreatment had the most severe consequences.” Indeed, history of emotional maltreatment in childhood has been similarly linked with maladaptive patterns of parental RF and less supportive caregiving behavior (Condon et al., 2021), consistent with the notion that experiencing one’s own caregiver as emotionally unavailable may play a role in impacting levels of RF in mothers with family history of maternal substance use.

Childhood experiences of maternal substance use and psychological unavailability may thus propagate on to impact the next generation’s parenting through maladaptive cognitions and behaviors (Milligan et al., 2022). Compromised RF capacities impedes one’s ability to make sense of their interpersonal relationships and trauma, both past and present (Fonagy et al., 1994; Berthelot et al., 2015). Furthermore, low parental RF often manifests in failing to see how one’s experiences in childhood have shaped their relationships with their own children (Slade et al., 2007). Thus, the lower RF observed among mothers with substance use problems who also had a family history of maternal substance use may indicate difficulties in reorganizing childhood experiences as adults and lacking the psychological and interpersonal resources to “break the cycle.” This may then manifest repeating patterns characteristic of low RF and lack of emotional availability with their own child (Milligan et al., 2022), including difficulties tolerating child distress and interpreting the child’s intentions as inappropriately hostile (Rutherford et al., 2015), and exhibiting hostility toward the child while struggling to support them (Rosenblum et al., 2008; Zeegers et al., 2017; Condon et al., 2021). Such repetition of past patterns may be even more salient under stress, as high stress and arousal can reactivate representations of attachment trauma (Luyten and Fonagy, 2015), potentially reinforcing both increased substance use (Rutherford and Mayes, 2019) and use of maladaptive pre-mentalizing modes of parental RF (Rutherford et al., 2015).

The paradox in our results is the finding that exposure to maternal substance use alone was associated with lower RF than exposure to maternal substance use in the presence of maternal mental illness. It is possible that participants whose mothers experienced mental illness were more likely to have been identified by healthcare providers, child protective services, or family members as needing additional support, with the ultimate outcome of being removed from the home and placed in more supportive kinship or foster care settings that supported RF development. Based on addiction typology literature (Cloninger et al., 1996), it may also be possible that individuals with type II mothers (i.e., substance use onset < 25 years, more severe addiction, polysubstance use) may have incurred more risk in early childhood associated with the earlier onset of their mothers’ substance use. In contrast, participants with type I parents (i.e., later onset of substance use, secondary to mental illness) may have experienced less risk in infancy and early childhood given that their mothers may have been more psychologically available before the onset of their addictions, therefore providing an early window for RF development.

Alternatively, participants who reported both maternal substance use and mental illness may have developed RF as an adaptive strategy for making sense of their mother’s substance, by understanding it as a means of coping with mental illness, for example. The developmental capacity of RF requires the ability to consider nuanced meaning driving a persons’ behavior, and it is possible that witnessing substance use in the context of maternal mental illness provided organization to the meaning-making process compared to children of mothers without mental illness. The context of maternal mental illness may also encourage an adaptive strategy where children grow attuned to (or even hypervigilant of) their mother’s affective dysregulation and become practiced at reflecting on and anticipating the mental states potentially underlying their mother’s behavior (Miller, 2008). This interpretation is in line with prior findings that children who experienced physical abuse (i.e., trauma accompanied by parental emotion dysregulation, inconsistency, and hostility) were highly adept at detecting subtle emotional cues (Pollak and Sinha, 2002). Similarly heightened emotion recognition and threat detection has been observed in children exposed to parental depression (Lopez-Duran et al., 2013) and substance use (Flykt et al., 2021). Overall, such hypersensitivity may help individuals predict and thereby cope with inconsistent, unpredictable, dysregulated, or confusing caregiving behavior exhibited by mothers experiencing mental illness. Though potentially adaptive in the context of one’s family of origin, it is unclear whether this heightened sensitivity to and reflection about others’ emotions translates to accurate detection of infant cues, and ultimately whether it is adaptive when caring for the next generation. It should also be kept in mind that despite the relatively higher level of RF in this group, mothers nonetheless exhibited below-average RF indicative of having some mentalizing difficulties.

### Limitations and future directions

Large clinical datasets like the one used in the current study lend valuable information to our understanding of factors affecting high-risk samples and the nuanced differences in RF that exist within such samples. Overall, this secondary data analysis requires replication and extension to further elucidate the links between risk factors for attachment trauma and RF in mothers, as well as fathers and other types of caregivers, with addictions. Although the overall sample was large, some potentially meaningful effects may not have proven significant because of limited statistical power associated with small cell size. The overall mean RF in our sample was also relatively low regardless of family history, and the statistically significant differences found in the current study may not necessarily translate to clinical significance. Given the nature of our secondary data analysis, we were also limited to the information collected as part of the broader RCTs investigating treatment efficacy. While we utilized a well-validated, widely used interview measure of parental RF (the PDI), our assessment of specific risk factors for attachment trauma was limited to mothers’ self-report on ASI items regarding the presence or absence of substance use and psychiatric problems in biologically-related family members. Due to the nature of the ASI, we unfortunately did not have details regarding severity or age of onset of parental mental illness/substance. Parental mental illness was also collapsed into one category which did not allow for subanalyses of the potential impact of different types of parental mental illness (e.g., depression vs. anxiety vs. personality pathology) on mothers’ RF. Future studies should employ a more detailed assessment of risk factors for attachment trauma including family history of mental illness and substance use in addition to other challenges that may interfere with a caregiver’s capacity to hold their child in mind such as intimate partner violence, poverty, and racism, amongst others. Alternatively, future research may investigate these relationships using a more detailed measure of unresolved trauma or hostile-helpless states of mind *via* the Adult Attachment Interview (Lyons-Ruth et al., 2003; Main et al., 2008). It will also be important to investigate whether our results are generalizable to other samples of mothers with addictions, including mothers who are not currently in substance use treatment, or mothers who are in residential or inpatient treatment. While the majority of mothers in our sample were in treatment for an opioid use disorder (MOUD), it may also be important to see if these results vary by primary drug of abuse. Future research examining the links between attachment trauma and RF may also include a measure of mothers’ other resiliency factors, such as participation in prior mental health treatment or other positive relational experiences (Lieberman et al., 2005; Narayan et al., 2017), that may have influenced their reflective capacities. In light of the findings of the current study, it may also be critical to examine whether family history of maternal mental illness and substance use may moderate mothers’ response to parenting interventions.

### Implications

Overall, studying mothers’ experiences within attachment relationships can enhance the field’s understanding of potential antecedents of reflective functioning in the context of addiction. Our findings highlight the importance of disentangling the contributions of different types of childhood adversity or attachment trauma, namely family history of mental illness and substance use, to mothers’ reflective capacities. Critically, a mother’s experience of her caregivers has the potential to influence the care she provides to the next generation. Treatment efforts may potentially be enhanced if we are able to identify underlying factors connecting maternal addiction with RF. Our findings suggest that clinicians working with mothers in recovery from substance use disorders might consider assessing family history of mental illness and substance use and utilizing this information in conceptualization, treatment planning, and clinical approach. Consistent with theoretical and empirical work (Suchman et al., 2004, 2006, 2010b), parenting interventions for mothers with addictions are most successful when there is a focus on enhancing mothers’ RF. Based on the results presented here, this may specifically include helping mothers reflect upon how their early experiences of being cared for by a mother with a mental illness and/or addiction may influence their current caregiving relationships, capacities, and behaviors.

## Data availability statement

The data analyzed in this study is subject to the following licenses/restrictions: Deidentified clinical baseline dataset collected as part of three randomized controlled trials. Requests to access these datasets should be directed to TM, thomas.mcmahon@yale.edu.

## Ethics statement

The studies involving human participants were reviewed and approved by Yale University Human Investigation Committee. The patients/participants provided their written informed consent to participate in this study.

## Author contributions

AL was responsible for the conceptualization and first draft of this manuscript, with contributions from TY, EP-C, AZ, AL, CD, and TM on subsequent drafts. TY and TM contributed to statistical analyses and interpretation. EP-C, AZ, CD, and TM contributed to interpretation of the findings and the editorial process. We posthumously acknowledge NS for having completed the three randomized controlled trials that supplied the baseline data included in this manuscript. All authors have approved the final submitted version of the manuscript.
